# The Multifaceted Roles of MicroRNAs in Cystic Fibrosis

**DOI:** 10.3390/diagnostics10121102

**Published:** 2020-12-17

**Authors:** Fatima Domenica Elisa De Palma, Valeria Raia, Guido Kroemer, Maria Chiara Maiuri

**Affiliations:** 1 Equipe 11 Labellisée Ligue Contre le Cancer, Centre de Recherche des Cordeliers, INSERM UMRS 1138, Sorbonne Université, Université of Paris, 75006 Paris, France; kroemer@orange.fr; 2 Metabolomics and Cell Biology Platforms, Gustave Roussy Comprehensive Cancer Institute, 94805 Villejuif, France; 3 CEINGE-Biotecnologie Avanzate, 80145 Naples, Italy; 4 Pediatric Unit, Department of Translational Medical Sciences, Regional Cystic Fibrosis Center, Federico II University Naples, 80131 Naples, Italy; raia@unina.it; 5 Suzhou Institute for Systems Medicine, Chinese Academy of Sciences, Suzhou 215123, China; 6 Karolinska Institutet, Department of Women’s and Children’s Health, 17176 Stockholm, Sweden; 7 Pôle de Biologie, Hôpital Européen Georges Pompidou, AP-HP, 75015 Paris, France; 8 Institut Universitaire de France, 75005 Paris, France

**Keywords:** cystic fibrosis, genetic disease, microRNA, circulating miRNAs, microRNA-targeted therapies, miRNA mimics, antimiRs, antagomiRs, biomarkers

## Abstract

Cystic fibrosis (CF) is a lifelong disorder affecting 1 in 3500 live births worldwide. It is a monogenetic autosomal recessive disease caused by loss-of-function mutations in the gene encoding the chloride channel cystic fibrosis transmembrane conductance regulator (CFTR), the impairment of which leads to ionic disequilibria in exocrine organs. This translates into a chronic multisystemic disease characterized by airway obstruction, respiratory infections, and pancreatic insufficiency as well as hepatobiliary and gastrointestinal dysfunction. Molecular characterization of the mutational heterogeneity of CFTR (affected by more than 2000 variants) improved the understanding and management of CF. However, these CFTR variants are linked to different clinical manifestations and phenotypes, and they affect response to treatments. Expanding evidence suggests that multisystemic disease affects CF pathology via impairing either CFTR or proteins regulated by CFTR. Thus, altering the expression of miRNAs in vivo could constitute an appealing strategy for developing new CF therapies. In this review, we will first describe the pathophysiology and clinical management of CF. Then, we will summarize the current knowledge on altered miRNAs in CF patients, with a focus on the miRNAs involved in the deregulation of CFTR and in the modulation of inflammation. We will highlight recent findings on the potential utility of measuring circulating miRNAs in CF as diagnostic, prognostic, and predictive biomarkers. Finally, we will provide an overview on potential miRNA-based therapeutic approaches.

## 1. Introduction

The dual role of time, as a fearsome enemy or important ally in the course of human diseases, has motivated researchers to advance in the frantic (and necessary) understanding of their cellular and molecular bases, hoping to identify novel approaches for patient care. The discovery of a variety of disease-modulatory molecules, including non-coding RNAs, that are present not only at the tissue level, but also in the bloodstream, has revolutionized the world of clinical molecular biology.

microRNAs (miRNAs), a class of small non-coding RNAs, are well known to regulate a diverse array of biological processes, such as proliferation, development, and metabolism [1,2]. They act as repressors of one or several genes by binding to complementary sites within the 3’ untranslated region (UTR) of target mRNAs [1,3,4]. 

miRNAs positively or negatively impact on the pathogenesis and/or progression of human diseases. They are (de)regulated by different mechanisms, such as epigenetic alterations, chromosomal abnormalities, as well as changes in transcriptional control [2,5]. Moreover, the altered abundance of miRNAs, along with their high stability (due to their small length of about 18–22 nucleotides) renders them highly potential biomarker candidates in non-invasive sources (i.e., blood, serum, urine, and saliva) [6,7]. 

The clinical potential of miRNAs as diagnostic and prognostic, as well as predictive biomarkers, has been widely described, especially in the context of human cancer [6,8,9,10]. Moreover, potential therapeutics harness the deregulation of miRNAs in disease conditions by means of miRNA mimics, so-called antimiRs [11,12].

Accumulating evidence suggests the involvement of miRNAs also in genetic diseases, including cystic fibrosis (CF) [13,14,15,16,17,18,19]. In particular, many studies showed the direct or indirect regulatory impact of deregulated miRNAs on the expression of the *cystic fibrosis transmembrane conductance regulator* (*CFTR*) mRNA [20]. Moreover, their participation in the control of the inflammation of CF airways has been demonstrated, along with their potential role as circulating biomarkers [17]. Thus, the contribution of miRNAs to disease progression and severity opens new and interesting scenarios for the clinical management of CF patients.

In this review article, we will describe the pathogenesis of CF, followed by an overview of the wide mutational spectrum harbored by the *CFTR* gene that translates into the large range of CF clinical phenotypes. CF care is a race against time and against the progressive and aggressive degeneration of multiple organs. Considering the benefits that non-coding RNAs could potentially offer for the treatment of human diseases, we cover current knowledge on the impact of miRNAs on CF. Thus, we will discuss the biological regulatory role of miRNAs in the progression of the disease. Moreover, we will describe circulating miRNAs as potential non-invasive biomarkers (i.e., for monitoring lung disease progression), as well as the role of single nucleotide polymorphisms (SNPs) associated with miRNAs in modulating CF phenotypes. Finally, we will summarize the therapeutic potential of miRNAs in CF.

## 2. Cystic Fibrosis 

### 2.1. Cystic Fibrosis, A Multisystemic Deadly Disease

CF, also known as mucoviscidosis, is one of the most common Mendelian autosomal recessive genetic disorders [21,22]. Its incidence varies according to ethnicity, affecting approximately 1 in about 2500 newborns in Europe [23]. 

CF is caused by mutations in *CFTR*, which belongs to the ATP binding cassette (ABC) gene family [22,24]. Environmental factors, as well as non-*CFTR* gene modifiers, influence the manifestations and progression of the disease [25].

The *CFTR* gene is located on the long arm of the chromosome 7 [25]. The CFTR protein is mostly located at the apical membranes of a variety of secretory ephitelia (which produce mucus, sweat, and digestive juices) harbored in multiple organs, including sweat glands, lungs, and pancreas, as well as the gut [21,25]. CFTR mainly functions as an anion channel; it acts as a cyclic adenosine monophosphate (cAMP)-dependent chloride channel and as bicarbonate channel. Moreover, the involvement of CFTR in the modulation of other ion channels, such as the epithelial sodium channel (ENaC), has been also described, not without controversy [26]. CFTR transports chloride and bicarbonate across secretory epithelia, thus regulating both the secretion and the absorption of salt and water and maintaining epithelial surface hydration. 

Mutations in *CFTR* lead to the impairment of the expression, as well as of the function and stability at the mRNA and CFTR protein levels [27], thus unbalancing fluid and electrolyte homeostasis. For instance, an abnormal excessive secretion of salt from the sweat glands that has not been reabsorbed by the sweat duct cells is one of the main symptoms of CF [25]. At present, the measurement of chloride excreted in sweat is the gold standard to diagnose CF. 

Another deleterious effect, which is due to the reduction of the bicarbonate release, is the formation of thickened and viscous secretions in bronchi, pancreas, biliary tract, intestine, and the reproductive system. Although mucus is an alley of innate immunity due to its composition made, inter alia, of antibacterial defensins and immunoglobulins, its hyperviscous form is pathogenic in CF [28]. 

Concerning the respiratory tract of CF patients, defects in fluid secretion and the reduced pH of the airway surface liquid affect ciliary beating and compromise mucociliary clearance, thus increasing the viscosity of the mucus but decreasing the activity of antimicrobial molecules [29]. As a consequence, mucus accumulates and obstructs the airways (bronchiectasis), promoting chronic bacterial infections and inflammatory lung damage. The airway microbiome changes across the ages [30]. Typically, CF adults are persistently colonized by *Pseudomonas aeruginosa* (*P. aeruginosa*) [31]. Altogether, these changes trigger acute pulmonary exacerbations and respiratory failure. 

The gastrointestinal tract as well as the biliary ducts are also impacted by unbalanced ion secretion [32,33]. The more acidic intestinal environment, along with the sticky and thickened mucus, which is hardly cleared, alters the activity of digestive enzymes; this impairs the assimilation of digestive products, delays the intestinal transit, and causes bowel obstructions [32]. Meconium ileus is one of the earliest clinical manifestations and the most serious acute complication of CF at the intestinal level [34]. Nonetheless, older CF patients may also present distal intestinal obstruction syndrome (DIOS) and intussusception [33]. Furthermore, intestinal inflammation and dysbiosis occur in the context of CF [32,35]. 

Severe inflammation, viscous secretions, and fibrosis lead to pancreatic insufficiency, leading to abdominal pain, malabsorption, and weight loss [36]. Additionally, diabetes mellitus and male infertility may become manifest [37,38,39]. Congenital aplasia of vas deferens (CAVD) contributes to male infertility and obstructive azoospermia [40,41]. This abnormality of the urogenital tract has different clinical presentations, according, inter alia, to its bilaterally (CBAVD) or unilaterally (CUAVD) occurrence [40,41,42]. In women with CF, fertility is compromised due to thicker cervical secretions [37]. 

Although CF is a chronic multisystemic disease that is ultimately lethal, daily care and personalized (expensive) treatments extend the life expectancy of most CF patients until 40 years of age [43]. The medical care for CF requires multiple interventions including high-calorie ingestion, therapy based on the replacement of pancreatic enzymes and, most importantly, the management of the pulmonary exacerbations [21,25,44]. For instance, airway-clearance techniques and aerosolized mucolytic agents, along with administration of some specific antibiotics and anti-inflammatory drugs can avoid, reduce, or eradicate bacterial infections and improve lung functions [25]. 

In addition, targeted therapies for CF patients with specific *CFTR* pathogenic mutations have been developed [43,44,45,46]. These treatments are based on a class of compounds, the CFTR modulators, which are able to modulate (enhance or restore) the expression, function, and stability of mutant CFTR proteins [43,45]. Generally, CFTR modulators are grouped into five classes: (i) “potentiators”, which restore the channel gating and conductance of the mutant CFTR protein; (ii) “correctors”, which are drugs that rescue the protein folding, thus augmenting the traffic to the plasma surface of CFTR mutant; (iii) “stabilizers” that avoid the removal and degradation by lysosomes of CFTR through its stronger stabilization on the plasma membrane; (iv) “amplifiers” that increase the expression of CFTR at the mRNA level, and, in turn, the amount of CFTR protein; and (v) “read-through” agents that restore the functional full-length protein affected by premature termination codons. To date, only two classes of modulators, potentiators and correctors, have been approved to treat CF patients [45].

Finally, lung transplantation is reserved for end-stage CF patients, but it extends survival by only 5 years on average [47]. 

### 2.2. The Mutational Landscape of the Cystic Fibrosis

Since the discovery of the first *CFTR* mutation in 1989, to date, more than 2000 different genetic variants have been identified (www.CFTR2.org). However, only a few of them (about 350 mutations) are considered as pathogenic (www.CFTR2.org) [43]. 

According to the traditional classification system, mutations harbored by *CFTR* are grouped into six classes, depending on their effect on the synthesis, function, or stability of the CFTR protein (Table 1) [27,44].

Specific mutations in *CFTR* relatively influence the severity of the disease and, therefore, translate into different clinical manifestations or CF phenotypes (Table 1) [27]. However, the severity of the disease is also related to the heterozygosity or homozygosity of the CFTR mutation [48]. The molecular diagnosis of *CFTR* mutations guides clinicians with regard to the management of the disease. 

Severe mutations belonging to classes I, II, and III (Table 1) [27] lead to the loss of the CFTR function. In particular, class I mutations abolish the synthesis of the protein. They include nonsense mutations (i.e., premature termination codons). Class II mutations cause the retention of a misfolded protein at the endoplasmic reticulum and its subsequent degradation by the proteasome. The most common mutation in Europe, the F508del, which consists in the deletion of a phenylalanine at position 508, belongs to this class. Class III mutations affect the regulation and gating of the CFTR channel. 

By contrast, class IV to VI mutations confer a milder CF phenotype (Table 1) [27]. Class IV mutations decrease the conductance of chloride and bicarbonate ions. Class V mutations lead to a reduction of the abundance (synthesis or maturation) of the normal CFTR protein. Class VI mutations destabilize the protein at the cell surface by increasing the endocytosis and lysosomal degradation of CFTR. 

A new categorization of CFTR mutations into VII classes according to the therapeutic strategies has been suggested by De Boeck and Amaral [46]. The only difference compared to the traditional classification system consists in the addition of class VII mutations, which have the same functional characteristics as class I mutations but cannot be rescued by pharmacological interventions [46]. 

## 3. microRNAs in Cystic Fibrosis

### Altered microRNAs in the Regulation of CFTR 

One of the first studies on miRNAs in CF employed transcriptomic and miRNA array-based approaches to show that altered levels of miRNAs correlated with the decreased expression of CFTR under hypoxic conditions [49]. Likewise, in silico predictions, followed by in vitro validations, recently described miR-200b as a potential negative regulator of CFTR mRNA levels in human airway epithelia during hypoxia [50].

Over the years, different groups showed that a variety of miRNAs repress directly (i.e., miR-101, miR-145, miR-223, miR-494, miR-509-3p and miR-1246) or indirectly (i.e., miR-9 and miR-138) the expression of CFTR [51,52,53,54,55,56,57,58]. Concerning this latter point, miRNAs can bind to the 3’ UTR of genes that modulate CFTR. For instance, miR-138 reportedly downregulates CFTR via targeting the transcriptional repressor gene *switch-independent 3 homolog A* (*SIN3A*) [58]. Another example concerns miR-9, the upregulation of which impaired the expression and activity of the gene *anoctamin 1* (*ANO1*) in bronchial epithelial cells, thus contributing to CF lung pathology [57]. *ANO1*, also known as calcium-activated chloride channel (TMEM16A), may be involved in the activation and membrane expression of CFTR [59].

By contrast, Gillen and collaborators demonstrated that miR-145 and miR-494 directly target the 3’ UTR of *CFTR* mRNA [54]. Interestingly, miR-384, miR-494, and miR-1246 are also implicated in the regulation of chloride channel transport by regulating the gene *solute carrier family (SLC) 12 member 2* (*SLC12A2*) [51]. Other investigations confirmed the deregulation of miR-101, miR-145, and miR-494 in different specimens from CF patients [51,52,53]. miR-101 was also found to be upregulated in the lung of patients with chronic obstructive pulmonary disease; this condition is characterized by low CFTR protein levels [52].

Concerning the involvement of the well-known miR-145 in the dysregulation of CFTR, a variety of mechanisms have been identified. For instance, elevated levels of miR-145 were found in CF versus non-CF nasal epithelial tissues and inversely correlated with the expression of *SMAD family member 3* (*SMAD3*). Furthermore, miR-145, along with miR-494 and miR-223, was increased in bronchial brushing from CF patients bearing the F508del mutation. Functional experimentation showed that the corresponding premiRs downregulated and antimiRs upregulated CFTR gene and protein expression. Moreover, defective chloride ion conductance, inflammatory and infective insults altered the levels of miR-145, miR-223, and miR-494 [60]. Recently, miR-145-5p and miR-223-3p were found to be overexpressed in F508del homozygous CF bronchial epithelial cells and specimens from children and adults with CF. High levels of these miRNAs were also found in CF and CFTR gene-corrected induced pluripotent stem cell-derived CF lung organoids. Additional studies provided evidence that the upregulation of miR-145 in CF might be partially secondary to the increased expression of transforming growth factor-β (TGF-β) [61,62].

CF and chronic bronchitis, as well as tobacco smokers, present a similar lung inflammation [63]. In this context, it has been demonstrated that air pollution and cigarette smoke (CS) play an important role in the progression of airway diseases by promoting CFTR dysfunction through miRNA deregulation. For instance, CS increased the expression of miR-101 that consequently reduced CFTR in human bronchial epithelial cells and in vivo [52]. Moreover, altered levels of miR-145-5p were involved in the TGF-β-mediated suppression of CFTR and the chloride channel, SLC26A9 [64]. Additionally, an antagomiR specific for miR-145-5p rescued CFTR and SLC26A9 expression in vitro, while the use of a neutralizing aptamer against another target of miR-145-5p, namely TGFBR2, was able to restore the suppression of CS- and TGF-β-mediated CFTR function in vivo [64]. 

Defective autophagy plays a critical role in the pathology of CF [65,66]. Therefore, the role of deregulated miRNAs in impairing the autophagy machinery in CF has been explored. Indeed, high levels of the cluster mirc1/mir17-92 contributed to the negative regulation of autophagy and CFTR function in CF macrophages [67].

## 4. microRNAs as Regulators of Inflammation in Cystic Fibrosis

**Figure 1:**
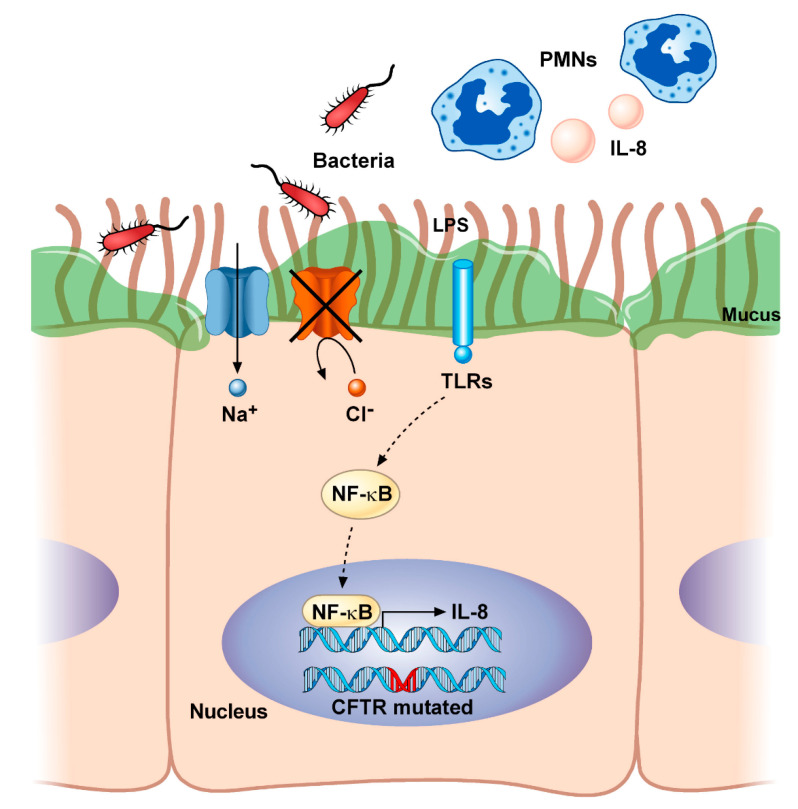
Airway inflammation in cystic fibrosis. Mutations in *CFTR* lead to imbalances in fluid and electrolyte homeostasis characterized by the lack of transport of chloride (Cl^−^) and excessive sodium (Na^+^) reabsorption. This impairs mucociliary clearance and increases the viscosity of the mucus, thus promoting chronic bacterial colonization (i.e., by *Staphylococcus aureus* and *Pseudomonas aeruginosa*). The resultant shedding of microbial molecules, known as pathogen-associated molecular patterns (PAMPs) (i.e., lipopolysaccharide, LPS), being recognized by the Toll-like receptors (TLRs), constitutively activates the NF-κB and causes the consequent production of inflammatory cytokines (i.e., IL-8), as well as the accumulation of polymorphonuclear neutrophils (PMNs) into the airways. CFTR, cystic fibrosis transmembrane conductance regulator; IL-8, interleukin-8; LPS, lipopolysaccharide; NF-κB, nuclear factor κ light-chain enhancer of activated B cells; PMNs, polymorphonuclear neutrophils; TLRs, toll-like receptors.

Inflammation plays a critical role in the progression of CF [68]. As described before, the overproduction of hyperviscous mucus obstructs airways and impairs mucociliary clearance. This creates a perfect nutrient-rich environment for bacterial colonization as well as for pro-inflammatory modulators that lead to the progressive structural damage of the lung (Figure 1). 

miRNAs can contribute to the maintenance of a pro- (mainly) or anti-inflammatory phenotype in CF patients via different ways [20]. Thus, the modulation of the abundance of miRNAs involved in these pathways may prevent lung disease and offer clinical applications in the future.

Essential fatty acid-derived lipoxins (LX), resolvins (Rv), protectins, and maresins are lipid mediators that control several aspects of the acute inflammation and resolution via specific G protein-coupled receptors (GPCRs) [69]. LXA_4_ and RvD_1_ activate a specific GPCR termed lipoxin A4 receptor (ALX)/formyl peptide receptor 2 (FPR2), which signals to inhibit NF-κB activation [69,70]. 

The role of the ALX/FPR2 receptor has been explored in relationship with CF. Interestingly, the ALX/FPR2-dependent pathway of inflammation resolution was altered by the overexpression of miR-181b in CF respiratory cells and macrophages [71].

It has been demonstrated that a variety of altered miRNAs are able to regulate the expression of several pro-inflammatory messengers, including interleukin (IL)-6 and IL-8. For instance, miR-146a upregulation is involved in CF inflammation. Thus, the elevation of miR-146a causes macrophages isolated from CF patients to overproduce IL-6 in response to lipopolysaccharide (LPS) stimulation [72]. IL-8 is produced by macrophages and bronchial epithelial cells in the CF lung in response to infectious (i.e., *Pseudomonas aeruginosa* and *Staphylococcus aureus*) and inflammatory (i.e., IL-1β or tumor necrosis factor-α, TNF-α) stimuli via different signaling pathways (i.e., NF-κB) [73,74]. The main function of IL-8 is to attract and activate neutrophils, which dominate the inflammatory response in the CF airway [74]. The secretion of IL-8 could be directly or indirectly impaired by deregulated miRNAs. For instance, IL-8 was identified as a direct target of miR-17 and miR-93 [75,76]. Particularly, low levels of miR-17 were found in CF cell lines, also when treated with *P. Aeruginosa*-conditioned medium, in CF bronchial brushings, and in βENaC-overexpressing (βENaC-Tg) mice with spontaneous airway neutrophilia, as well as in mucus obstructions [75]. miR-93 was identified as significantly downregulated in bronchial epithelial cells infected with *P. aeruginosa* through a microarray technique [76]. For both microRNAs, luciferase assays, along with other in vitro experiments, demonstrated that their deregulation directly increased the production of IL-8.

Concerning indirect modulations, high levels of miR-155 in CF cells inhibited the translation of the gene *SH-2 containing inositol 5’ polyphosphatase 1* (*SHIP1*), thus leading to the activation of the signaling pathway mediated by phosphatidylinositol-3 kinase/protein kinase B (PI3K/AKT) and the consequent secretion of IL-8 [77]. The same authors showed that the expression of miR-155 in CF was suppressed by tristetraprolin (TTP) through the induction of miR-1 but enhanced by KH-type splicing regulatory protein (KSRP) via promoting its maturation in vitro [78]. Moreover, miR-155 may also regulate the fibrosis of CF lungs. In fact, it can suppress the expression of regulatory associated protein of mTOR complex 1 (RPTOR), thus consequently increasing the abundance of connective tissue growth factor (CTGF) in CF lung epithelial cells [79].

Recently, miRNome and transcriptomic analyses showed that miR199a-3p was less expressed in CF bronchial explants extracted from CF patients than in non-CF individuals [80]. Moreover, wet-lab experimentations revealed that the downregulation of miR-199a-3p was associated with the hyperactivation of the NF-κB pathway and, therefore, with the increased secretion of IL-8 via targeting the inhibitor of nuclear factor kappa-B kinase subunit beta (IKKβ) [80].

miRNAs can also control other signaling pathways to trigger inflammation in CF. High levels of miR-199a-5p were detected in human and murine CF macrophages and murine CF lungs [81]. The aberrant expression of miR-199a-5p, mediated by PI3K/AKT signaling, reduced the expression of caveolin 1 (CAV1), which is a mediator of inflammation processes, and in turn increased Toll-like receptor (TLR) 4. Moreover, the downregulation of miR-199a-5p was able to reduce the inflammation in CF macrophages via restoring the expression of CAV1. Furthermore, in vitro and in vivo studies demonstrated that the administration of the non-steroidal anti-inflammatory drug celecoxib rescued the altered pathway mediated by AKT/miR-199a-5p/CAV1 in CF macrophages and reduced lung inflammation in *CFTR*-deficient mice, respectively [81].

An elevated expression of miR-145, found in nasal airway cells from CF patients when compared to non-CF controls, correlated with the downregulation of SMAD3 [82]. SMAD3 is a negative modulator of the NF-kB–IL-8 pathway mediated by TGF-β1; therefore, miR-145 may contribute to CF-related inflammation [82]. 

Differently from the aforementioned miRNAs, miR-126 has an anti-inflammatory role in CF. It has been the first miRNA identified as deregulated in CF in 2010 [83]. In particular, low levels of miR-126 were found in CF airway epithelial cells. This downregulation correlated with the upregulation of the target of Myb protein 1 (TOM1), which is a negative regulator of TLR2, TLR4, IL-1, IL-1β, TNF-α, and NF-kB. When CF cells were stimulated with LPS and IL-1β, the knockdown of TOM1 significantly increased the NF-kB-mediated IL-8 secretion [83]. In addition, another study reported that miR-1343 attenuated the pathway of fibrosis by decreasing levels of activated TGF-β effector molecules, including phosphorylated (p) SMAD3 (pSMAD3) as well as pSMAD2 and consequently disturbing the cell migration and epithelial–mesenchymal transition [84].

Some miRNAs may also participate in chronic inflammation by affecting the remodeling of the pulmonary epithelium or by impacting the expression of genes involved in mucus hypersecretion. The role of miR-146a in CF inflammation has been linked to its negative impact on the production of the mucin 5AC (MUC5AC), which is a major component of the airway mucus [85]. Moreover, the knockdown of miR-146a in human bronchial epithelial cells resulted in the activation of the NF-kB and Jun N-terminal kinase (JNK) pathways [85].

In bronchial epithelial cells, miR-145, miR-494, and particularly miR-221 regulate the transcription factor 6 (ATF6), which is implicated in the airway inflammation through endoplasmic reticulum stress [86,87].

The deregulation of miR-31 enhanced the production of the cathepsin S (CTSS) via the direct inhibition of the interferon regulatory factor 1 (IRF-1) in CF epithelial cells [88]. CTSS is an elastinolytic and collagenolytic cysteine protease. High levels of CTSS in CF patients increased pulmonary neutrophilic infiltration of the lung, as well as the inactivation of antimicrobial proteins, such as lactoferrin and members of the β-defensin family, therefore contributing to lung inflammation and infection, and determining lung damage [89].

## 5. Circulating microRNAs as Potential Biomarkers in Cystic Fibrosis

miRNAs can be quantified in non-invasive specimens such as body fluids, which renders them easily detectable biomarkers [13,90,91,92]. Indeed, miRNAs are highly stable and circulate in the bloodstream as cell-free miRNAs or packaged inside microvesicles, such as exosomes [9,93]. Advances in liquid biopsy technologies (i.e., digital PCR and microfluidic single-cells technologies) enable their accurate detection [94]. 

Although newborn bloodspot screening (NBS) offers the opportunity of early diagnosis, CF is a pediatric disorder that most often begins as a silent disease. Thus, along with the clinical examination, the detection and the assessment of the levels of expression of a single specific miRNA or signature of miRNAs in easily obtained biological sources, could be beneficial for the care of patients affected by CF. The potential role of miRNAs as circulating CF biomarkers has been explored in a few studies. 

The Mirc1/Mir17–92 cluster has been identified as a potential biomarker for CF disease progression [95]. In particular, high levels of the Mirc1/Mir17–92 cluster in CF sputum correlated with pulmonary exacerbations, lung function, and age. 

Recently, a profiling study comparing plasma miRNAs in CF patients versus non-CF controls identified a total of 11 deregulated miRNAs. In particular, 10 miRNAs (miR-16-5p, miR-92a-3p, miR-103a-3p, miR-103b, miR-107, miR-191-5p, miR-3613-5p, let-7a-5p let-7b-5p, and hsa-let-7d-5p) were overexpressed, while only miR-598-3p was reduced in CF [96]. Furthermore, bioinformatics analyses revealed that the target genes of these miRNAs were enriched in transduction pathways, such as the mTOR and PI3K/AKT, as well as Wnt/β-catenin signaling pathways [96].

CF presents, inter alia, a gender dichotomy [97,98]. Women are affected by poorer lung function as well as lower median survival. To study this gender gap, circulating miRNAs were profiled in male and female pediatric CF plasma samples [99]. miR-885 was increased in samples from girls under six years. Moreover, in silico analyses suggested that the severity of the disease in CF females could be related to a Ras-related C3 botulinum toxin substrate 1 (RAC1)-mediated process. However, functional experiments need to validate these theoretical predictions [99]. 

The involvement of circulating miRNAs in CF-related diabetes (CFRD), as well as in CF-associated liver disease (CFLD), has also been explored [100,101,102,103]. 

CFRD is the major extra-pulmonary co-morbidity in CF patients [38]. It significantly accelerates lung damage, leading to early mortality. The deregulation of miR-146a has been documented in CFRD [103]. In particular, high levels of the circulating miR-146 in serum samples from CF patients correlated with the onset of the CFRD. In addition, another study reported a significant increase of miR-146 expression in peripheral blood mononuclear cells cultured with CF plasma [104].

CFLD is a complication of CF that affects up to 40% of CF patients [105,106,107]. CFLD has been attributed to ductal cholestasis [106]. The pathogenetic mechanism of CFLD has not been fully elucidated yet. Moreover, the diagnostic criteria, captained by invasive liver biopsy, as well as the methods to monitor the progression of the disease are still inadequate [108]. In this context, miRNAs could be useful biomarkers in the management of CFLD.

Changes in the levels of circulating miR-122, miR-21, and miR-25 were able to identify CF patients affected by liver disease [100]. In detail, miR-122 was more expressed in CFLD patients when compared to CF patients without liver symptoms, as well as to healthy individuals. Moreover, miR-21 and miR-25 were overexpressed in CF patients with liver fibrosis compared to CF patients without fibrosis or controls. Additionally, circulating miRNA levels were also able to stratify CFLD patients according to the hepatic fibrosis stage (F0 = no fibrosis; F1–F4 = any histological evidence of fibrosis; F3–F4 = severe fibrosis). In particular, the combination of six miRNAs (miR-122, miR-21, miR-25, miR-210, miR-148a, and miR-19a) identified CF patients with early liver fibrinogenesis (F0–F1) and differentiated CFLD children without (F0) from those with any liver fibrosis (F1–F4) [100]. 

In order to diagnose and assess CFLD severity, Calvopina and collaborators investigated the combined use of serum miRNAs and aspartate aminotransferase (AST) to platelet ratio (APRI) [102]. An miRNA sequencing approach identified the circulatory miRNA signature of a total of 124 children (CF and CFLD patients, and healthy controls). Among the miRNAs detected, CFLD patients presented high levels of miR-122-5p, miR-365-33p, and miR-34a-5p, while low levels of miR-142-3p and let-7g-5p were detected with respect to CF children without liver disease. Moreover, the combination of miR-365a-3p, miR-142-3p, and let-7g-5p with APRI was able to predict liver disease in CF patients with high specificity (83%) and sensitivity (92%). Furthermore, the abundance of miR-18a-5p in serum specimens discriminated CF patients presenting severe (F3-F4) from mild/moderate (F0-F2) fibrosis [102].

In summary, emerging evidence suggests that circulating miRNAs may contribute to the diagnosis of CF complications. However, it will be important to overcome technical limitations (i.e., in sample collection, processing, and data analysis) and to obtain cognitive insights into the disease-modulatory role of such miRNAs.

## 6. Single Nucleotide Polymorphisms (SNPs) in microRNAs Targeting the CFTR Gene

As described above, CF is characterized by a large clinical heterogeneity. The variability of CF phenotypes is further accentuated by SNPs in the 3’ UTR of miRNA-targeted genes, as demonstrated by Amato and colleagues [56]. In particular, the SNP rs10234329, which is characterized by an adenine to cytosine (A>C) base substitution, was identified within the 3′UTR of the *CFTR* gene in a patient with a CFTR-related disease. Interestingly, in silico analyses demonstrated that this SNP was located in the target site of two miRNAs, including miR-433 and miR-509-3p. Moreover, in vitro experiments showed that this SNP reduced the expression of CFTR [56]. Thus, the 3′ UTR region of *CFTR* should be examined in CF patients that present clinical symptoms but lack mutations in the exons coding for the CFTR protein. 

Another investigation identified polymorphisms in the region of a miRNA cluster (miR-99b/let-7e/miR-125a) by examining CF F508del patients [109]. In particular, two SNPs detected (rs376594280 and rs41275794) modulated the maturation and, therefore, altered the expression of the miR-99b/let-7e/miR-125a cluster. These results shed light on the variability of CF severity in patients bearing the same *CFTR* genotype. 

## 7. Therapeutic Modulation of microRNAs in Cystic Fibrosis

### 7.1. microRNA Therapeutics

The modulation of the abundance of miRNAs may constitute a therapeutic strategy [11,12,110]. This can be achieved by using miRNA mimics or antimiRs [11,12,111]. 

miRNA mimics are synthetic double-stranded small RNAs used to re-establish the concentration of a specific downregulated miRNA [11,12]. Inversely, antimiRs suppress the function of overexpressed miRNAs. Generally, the function of miRNAs can be inhibited by three different approaches: (i) small-molecule inhibitors, which comprise specific compounds that repress the expression of a specific target miRNA (i.e., azobenzene for miR-21); (ii) miRNA sponges (expression vectors), which are transcripts that contain multiple, tandem binding sites to a microRNA of interest [112,113], thus acting as decoys of the target miRNA; and (iii) antisense oligonucleotides (ASOs) that competitively block the binding of a miRNA to its mRNA target [11,111,114]. 

Oligonucleotides are vulnerable to degradation by RNases present in serum or inside cells, resulting in poor pharmacokinetics [111]. Therefore, it is necessary to enhance their stability and binding affinity through two specific complementary strategies [11,111]: (i) chemical strategies relying on sugar and RNA backbone modifications, such as the addition of 2’-O-methyl (2-O’-Me) or phosphorothioate-like groups, locked nucleic acids (LNA), morpholinos, or peptide nucleic acids (PNA); and (ii) delivery strategies (i.e., viral vectors, polymer- and lipid-based delivery systems) aiming to encapsulate miRNAs, thus favoring their nuclease resistance and endosomal escape. Overall, methylation is often used for the generation of miRNA mimics, while LNA is mostly chosen for antimiRs. However, for in vivo applications, the phosphorothioate substitution is preferable [12]. In fact, this latter is not only particularly resistant to nucleases but also enhances the binding affinity with plasma proteins, so being quickly absorbed into the bloodstream [12]. To date, oligonucleotides are only administered via intravenous infusion or subcutaneous injection [12].

In spite of the interest of pharmaceutical companies for miRNA therapeutics, a variety of challenges need to be overcome, including tissue-specific targeting, toxicities, and off-targets effects [12,115]. For instance, chemical modifications could lead to adverse toxic effects in vivo, such as immune cell activation, altered coagulation, as well as hepatotoxicity [12,111]. Moreover, accurate tissue-specific delivery strategies need to be developed to minimize the risk of side effects in normal tissues as well as to attenuate the immune response. 

The optimization of the design of miRNA-based drugs is another obstacle. Design factors include the size and lipophilicity of the molecules to ensure efficient adsorption by the target tissue but to avoid fast renal clearance [12].

### 7.2. microRNA Therapeutics in Cystic Fibrosis

Endogenous miRNAs may affect the expression of *CFTR*, thus exacerbating the symptoms of CF [15,17]. Thus, miRNA mimics and antimiR agents could represent an appealing therapeutic strategy [116,117]. However, because each miRNA may simultaneously target several mRNAs, this approach could lead to unpredictable off-target effects. Moreover, the tissue-specific delivery and the stability of miRNA mimics and antimiRs are critical hurdles for this approach. An example concerns the miR-138 mimic. As previously mentioned, miR-138 indirectly impairs CFTR through the downregulation of SIN3A [58]. This latter is a transcriptional repressor of CFTR. The manipulation of the expression of miR-138 was able to restore CFTR abundance, as well as the chloride channel permeability in CF bronchial epithelial cells via upregulating SIN3A [58]. Nevertheless, this strategy led to undesired side effects on other genes regulated by SIN3A. 

Polymeric nanoparticles may be used to effectively deliver miRNA mimics. The pro-inflammatory miR-126, the downregulation of which leads to the overexpression of TOM1 in CF airways, has been used as a proof-of-concept [118]. Cationic nanoparticles successfully delivered miR-126-mimics into CF cell lines, thus significantly decreasing the expression of TOM1.

PNAs are also considered a valid anti-miR strategy [111]. PNAs are DNA analogues in which the sugar–phosphate backbone of the nucleic acid has been replaced by a synthetic achiral peptide backbone [111]. PNAs may be used to target specific miRNAs that are involved in the regulation of the expression of the CFTR gene [119,120]. For instance, PNAs designed to target miR-145, miR-509-3p, and miR-101-3p restored the expression of CFTR in vitro [61,121,122,123]. Additionally, PNAs can also be used as miRNA target protectors. In particular, PNAs can increase the expression of CFTR by competitively inhibiting the interaction of miRNAs with their mRNA targets [124]. However, these PNA-mediated approaches may present off-target effects on other miRNAs as well as on other target genes [122,125].

Another strategy involves target site blockers (TSBs) [111]. TSBs are locked nucleic acid antisense oligonucleotides that may have comparatively few off-target effects [111]. Indeed, TSBs compete with miRNAs for the binding to the miRNA target site at an mRNA. TSBs have been used to target miR-101 and miR-145 in the CFTR 3′ UTR, thus increasing CFTR expression and function in CF nasal epithelial cells as well as correcting chloride efflux mediated by ANO1 [53,57]. Thus, TSBs were successfully designed to prevent the binding of miR-9 to the 3’ UTR of ANO1 mRNA [57]. Recently, De Santi and colleagues reverted the inhibition of CFTR mediated by miRNAs via CFTR-specific TSBs in CF bronchial epithelial cells [126]. In particular, TSBs targeting the binding sites of miR-223-3p and miR-145-5p in the 3’ UTR were encapsulated in poly-lactic-co-glycolic acid (PLGA) nanoparticles. Their delivery via nebulization, alone or in combination with CFTR modulators (ivacaftor/lumacaftor and ivacaftor/tezacaftor), effectively enhanced the protein levels of CFTR. Due to the high specificity of TSBs for CFTR and the biocompatibility of PLGA, this strategy has low off-targets, toxicity, and immunogenicity with respect to other approaches.

Manipulation of miRNAs may also be relevant to achieve F508del CFTR correction. As mentioned above, the upregulation of TGF-β increased the expression of miR-145, which consequently caused the inhibition of CFTR [61,62]. In addition, functional experiments revealed that high levels of miR-145 nullified the effects of the CFTR modulators. Thus, an antimiR strategy directed against miR-145 was able to enhance the benefits of F508del CFTR correction in CF airway epithelia.

## 8. Conclusions

Every CF patient is unique with respect to her or his clinical manifestations as a result of the heterogeneity of CFTR mutations. Over the past decade, major advances have been achieved in the comprehension and treatment of CF, thus allowing the implementation of personalized medicine. Patients are stratified according to their CFTR mutations, which determine the therapeutic strategy [43]. Nonetheless, the phenotypic variability in patients with the same CFTR genotype remains a major therapeutic challenge. Moreover, many rare pathogenic mutations still lack efficient therapeutic options. Future progress in CF research requires, inter alia, appropriate cellular and animal models. In vivo models (i.e., mice, pigs, and ferrets) are particularly useful to explore the pathophysiology of CF and therapeutic strategies [127]. Nevertheless, their use in research has many limitations and disadvantages [127]. CF murine models fail to spontaneously develop lung disease, as well as bacterial infections, due to their short life span [128]. Meanwhile, pigs, which are highly attractive candidates, are substantially more complicated to manage (i.e., facility spaces, food, and costs) compared to other models [128]. The advent of organoid technology provides a unique platform to study a variety of human disorders, including CF. Organoid models created from patient cells are an affordable and simple way to explore disease mechanisms and to evaluate drug effects [129]. For example, Dekkers and colleagues demonstrated that the forskolin-induced swelling of rectal organoids isolated from small endoscopic biopsies from CF patients varied according to the specific CFTR mutation [130]. 

Deregulated miRNAs have a significant impact on the clinical course of CF [117]. The altered abundance of miRNAs affects multiple aspects of CF pathology. Indeed, miRNAs directly modulate the expression of CFTR (as this is the case for miR-101, -145, -223, -494, and -509) or indirectly affect CF (as this is the case for miR-138) by regulating other channels or proteins that act downstream of CFTR. Moreover, miRNA deregulation critically contributes to the inflammatory process (the case for miR-126, 199a-5p) or facilitates airway obstruction by altering the composition of mucus (the case of miR-17 and miR-146). 

In human pathologies, miRNAs may serve as diagnostic, prognostic, and predictive biomarkers due to their intrinsic properties, such as stability, facile detection, and disease-specific expression in non-invasive specimens [92]. There are no reliable biomarkers to monitor CF progression. It is possible that circulating miRNAs will help with evaluating and predicting fluctuations in lung inflammations as well as treatment responses. Currently, an ongoing clinical trial is measuring miRNAs extracted from blood specimens in order to stratify CF patients affected by mild or severe lung disease (NCT02992080, clinicaltrials.gov). Thus far, only a few studies have attempted to identify miRNAs as CF biomarkers. Large validation cohorts will be necessary to confirm the preliminary results obtained in these studies.

Understanding miRNA deregulation, distribution, targets, and function could unravel the biological processes that drive CF pathogenesis and, in addition, prepare the grounds for therapeutic interventions on miRNAs. Due to their small size and low antigenicity, miRNAs constitute attractive pharmacological agents for regulating the abundance of target mRNAs/proteins and pathways of interest. Most miRNA-relevant strategies rely on the use of synthetic, sequence-specific molecules that mimic or repress the expression of miRNAs. Some studies have already identified interesting miRNA-based therapeutics (i.e., TSBs targeting the binding sites of miR-223-3p and miR-145-5p) that could be used either alone or in combination with CFTR modulators [126]. However, several challenges, which include safe and organ-specific delivery, long-term efficacy, as well as side effects of prolonged treatments, need to be overcome. Pending the resolution of these issues, it is reasonable to hope that miRNA-targeting agents will eventually be introduced into the clinics. 

## Figures and Tables

**Table 1:** Classes of mutations in the cystic fibrosis transmembrane conductance regulator (CFTR) gene according to the traditional classification system.

Class of Mutation	CFTR Molecular Defect	Functional Abnormal Consequence	Mutation Examples	Type of Mutations	Clinical Phenotype	Therapeutic Strategy
I	No mRNA and protein synthesis	Absent protein	G542X, R553X, W1282X	Nonsense, frameshift, canonical splicing	Severe	Read-through agents
II	Reduced protein processing and traffic	Misfolded protein	F508del, N1303K, I507del	Missense, aminoacid deletion,	Severe	Correctors
III	Impaired channel gating	Reduced or absent channel opening	S549N, G551D	Missense, aminoacid change	Severe	Potentiators
IV	Decreased channel conductance	Defect in ion transport	R347P, R117H, D1152H	Missense, amino acid change	Mild	Potentiators
V	Reduced protein synthesis	Decreased protein	3849 + 10 kb C>T, A455E	Splicing defect, missense	Mild	Potentiators, correctors, ASOs
VI	Less protein stability and protein turnover at cell surface	Decreased half-life of the protein	120del23, G1412X	Missense, aminoacid change	Mild	Stabilisers

ASOs, antisense oligonucleotides; CFTR, cystic fibrosis transmembrane conductance regulator.

## References

[1] Jonas S., Izaurralde E. (2015). Towards a molecular understanding of microRNA-mediated gene silencing. Nat. Rev. Genet..

[2] Peng Y., Croce C.M. (2016). The role of MicroRNAs in human cancer. Signal Transduct. Target. Ther..

[3] Cech T.R., Steitz J.A. (2014). The Noncoding RNA Revolution—Trashing Old Rules to Forge New Ones. Cell.

[4] Filipowicz W., Bhattacharyya S.N., Sonenberg N. (2008). Mechanisms of post-transcriptional regulation by microRNAs: Are the answers in sight?. Nat. Rev. Genet..

[5] Han L., Witmer P.D., Casey E., Valle D., Sukumar S. (2007). DNA methylation regulates MicroRNA expression. Cancer Biol..

[6] Hayes J., Peruzzi P.P., Lawler S. (2014). MicroRNAs in cancer: Biomarkers, functions and therapy. Trends Mol. Med..

[7] Condrat C.E., Thompson D.C., Barbu M.G., Bugnar O.L., Boboc A., Cretoiu D., Suciu N., Cretoiu S.M., Voinea S.C. (2020). miRNAs as Biomarkers in Disease: Latest Findings Regarding Their Role in Diagnosis and Prognosis. Cells.

[8] Lan H., Lu H., Wang X., Jin H. MicroRNAs as Potential Biomarkers in Cancer: Opportunities and Challenges. https://www.hindawi.com/journals/bmri/2015/125094/.

[9] De Palma F.D.E., Luglio G., Tropeano F.P., Pagano G., D’Armiento M., Kroemer G., Maiuri M.C., De Palma G.D. (2020). The Role of Micro-RNAs and Circulating Tumor Markers as Predictors of Response to Neoadjuvant Therapy in Locally Advanced Rectal Cancer. Int. J. Mol. Sci..

[10] De Palma F.D.E., D’Argenio V., Pol J., Kroemer G., Maiuri M.C., Salvatore F. (2019). The Molecular Hallmarks of the Serrated Pathway in Colorectal Cancer. Cancers.

[11] Rupaimoole R., Slack F.J. (2017). MicroRNA therapeutics: Towards a new era for the management of cancer and other diseases. Nat. Rev. Drug Discov..

[12] Li Z., Rana T.M. (2014). Therapeutic targeting of microRNAs: Current status and future challenges. Nat. Rev. Drug Discov..

[13] Finotti A., Fabbri E., Lampronti I., Gasparello J., Borgatti M., Gambari R. (2019). MicroRNAs and Long Non-coding RNAs in Genetic Diseases. Mol. Diagn..

[14] Narożna B., Langwiński W., Szczepankiewicz A. (2017). Non-Coding RNAs in Pediatric Airway Diseases. Genes.

[15] Greene C.M. (2013). MicroRNA Expression in Cystic Fibrosis Airway Epithelium. Biomolecules.

[16] Varilh J., Bonini J., Taulan-Cadars M. (2015). Role of Non-coding RNAs in Cystic Fibrosis. Cyst. Fibros. Light New Res..

[17] Sonneville F., Ruffin M., Guillot L., Rousselet N., Le Rouzic P., Corvol H., Tabary O. (2015). New insights about miRNAs in cystic fibrosis. Am. J. Pathol..

[18] Noel S., Leal T. (2015). Emerging Roles of microRNAs in Cystic Fibrosis—From Pathogenesis to Development of New Therapies. Cyst. Fibros. Light New Res..

[19] Mitash N., Donovan J.E., Swiatecka-Urban A. (2020). The Role of MicroRNA in the Airway Surface Liquid Homeostasis. Int. J. Mol. Sci..

[20] McKiernan P.J., Greene C.M. MicroRNA Dysregulation in Cystic Fibrosis. https://www.hindawi.com/journals/mi/2015/529642/.

[21] Elborn J.S. (2016). Cystic fibrosis. Lancet.

[22] Cutting G.R. (2015). Cystic fibrosis genetics: From molecular understanding to clinical application. Nat. Rev. Genet..

[23] Scotet V., L’Hostis C., Férec C. (2020). The Changing Epidemiology of Cystic Fibrosis: Incidence, Survival and Impact of the CFTR Gene Discovery. Genes.

[24] Hwang T.-C., Kirk K.L. (2013). The CFTR Ion Channel: Gating, Regulation, and Anion Permeation. Cold Spring Harb. Perspect. Med..

[25] Ratjen F., Bell S.C., Rowe S.M., Goss C.H., Quittner A.L., Bush A. (2015). Cystic fibrosis. Nat. Rev. Dis. Primers.

[26] Saint-Criq V., Gray M.A. (2017). Role of CFTR in epithelial physiology. Cell. Mol. Life Sci..

[27] Marson F.A.L., Bertuzzo C.S., Ribeiro J.D. (2016). Classification of CFTR mutation classes. Lancet Respir. Med..

[28] Puchelle E., Bajolet O., Abély M. (2002). Airway mucus in cystic fibrosis. Paediatr. Respir. Rev..

[29] Chioccioli M., Feriani L., Kotar J., Bratcher P.E., Cicuta P. (2019). Phenotyping ciliary dynamics and coordination in response to CFTR-modulators in Cystic Fibrosis respiratory epithelial cells. Nat. Commun..

[30] Françoise A., Héry-Arnaud G. (2020). The Microbiome in Cystic Fibrosis Pulmonary Disease. Genes.

[31] Coburn B., Wang P.W., Diaz Caballero J., Clark S.T., Brahma V., Donaldson S., Zhang Y., Surendra A., Gong Y., Elizabeth Tullis D. (2015). Lung microbiota across age and disease stage in cystic fibrosis. Sci. Rep..

[32] Ooi C.Y., Durie P.R. (2016). Cystic fibrosis from the gastroenterologist’s perspective. Nat. Rev. Gastroenterol. Hepatol..

[33] De Lisle R.C., Borowitz D. (2013). The cystic fibrosis intestine. Cold Spring Harb. Perspect. Med..

[34] Bagolan P., Morini F., Conforti A., Puri P. (2020). Meconium Ileus. Pediatric Surgery: General Principles and Newborn Surgery.

[35] Hayden H.S., Eng A., Pope C.E., Brittnacher M.J., Vo A.T., Weiss E.J., Hager K.R., Martin B.D., Leung D.H., Heltshe S.L. (2020). Fecal dysbiosis in infants with cystic fibrosis is associated with early linear growth failure. Nat. Med..

[36] Madácsy T., Pallagi P., Maleth J. (2018). Cystic Fibrosis of the Pancreas: The Role of CFTR Channel in the Regulation of Intracellular Ca2+ Signaling and Mitochondrial Function in the Exocrine Pancreas. Front. Physiol..

[37] Casciaro R., Cresta F., Favilli F., Minicucci L. (2015). Cystic Fibrosis and Fertility. Cyst. Fibros. Light New Res..

[38] Kayani K., Mohammed R., Mohiaddin H. (2018). Cystic Fibrosis-Related Diabetes. Front. Endocrinol..

[39] Wilson C. (2011). Cystic fibrosis-related diabetes | Nature Reviews Endocrinology. Nat. Rev. Endocrinol..

[40] Bieth E., Hamdi S.M., Mieusset R. (2020). Genetics of the congenital absence of the vas deferens. Hum. Genet..

[41] de Souza D.A.S., Faucz F.R., Pereira-Ferrari L., Sotomaior V.S., Raskin S. (2018). Congenital Bilateral Absence of the Vas Deferens as an Atypical Form of Cystic Fibrosis: Reproductive Implications and Genetic Counseling. Andrology.

[42] Wagenknecht L.V., Lotzin C.F., Sommer H.-J., Schirren C. (1983). Vas Deferens Aplasia: Clinical and Anatomical Features of 90 Cases. Andrologia.

[43] Lopes-Pacheco M. (2020). CFTR Modulators: The Changing Face of Cystic Fibrosis in the Era of Precision Medicine. Front. Pharm..

[44] Maiuri L., Raia V., Kroemer G. (2017). Strategies for the etiological therapy of cystic fibrosis. Cell Death Differ..

[45] Clancy J.P., Cotton C.U., Donaldson S.H., Solomon G.M., VanDevanter D.R., Boyle M.P., Gentzsch M., Nick J.A., Illek B., Wallenburg J.C. (2019). CFTR modulator theratyping: Current status, gaps and future directions. J. Cyst. Fibros..

[46] Boeck K.D., Amaral M.D. (2016). Progress in therapies for cystic fibrosis. Lancet Respir. Med..

[47] Ramos K.J., Smith P.J., McKone E.F., Pilewski J.M., Lucy A., Hempstead S.E., Tallarico E., Faro A., Rosenbluth D.B., Gray A.L. (2019). Lung transplant referral for individuals with cystic fibrosis: Cystic Fibrosis Foundation consensus guidelines. J. Cyst. Fibros..

[48] Fisman D. (2020). Cystic fibrosis heterozygosity: Carrier state or haploinsufficiency?. Proc. Natl. Acad. Sci. USA.

[49] Guimbellot J.S., Erickson S.W., Mehta T., Wen H., Page G.P., Sorscher E.J., Hong J.S. (2009). Correlation of microRNA levels during hypoxia with predicted target mRNAs through genome-wide microarray analysis. BMC Med. Genom..

[50] Bartoszewska S., Kamysz W., Jakiela B., Sanak M., Króliczewski J., Bebok Z., Bartoszewski R., Collawn J.F. (2017). miR-200b downregulates CFTR during hypoxia in human lung epithelial cells. Cell. Mol. Biol. Lett..

[51] Megiorni F., Cialfi S., Dominici C., Quattrucci S., Pizzuti A. (2011). Synergistic post-transcriptional regulation of the Cystic Fibrosis Transmembrane conductance Regulator (CFTR) by miR-101 and miR-494 specific binding. PLoS ONE.

[52] Hassan F., Nuovo G.J., Crawford M., Boyaka P.N., Kirkby S., Nana-Sinkam S.P., Cormet-Boyaka E. (2012). MiR-101 and miR-144 Regulate the Expression of the CFTR Chloride Channel in the Lung. PLoS ONE.

[53] Viart V., Bergougnoux A., Bonini J., Varilh J., Chiron R., Tabary O., Molinari N., Claustres M., Taulan-Cadars M. (2015). Transcription factors and miRNAs that regulate fetal to adult CFTR expression change are new targets for cystic fibrosis. Eur. Respir. J..

[54] Gillen A.E., Gosalia N., Leir S.-H., Harris A. (2011). MicroRNA regulation of expression of the cystic fibrosis transmembrane conductance regulator gene. Biochem. J..

[55] Ramachandran S., Karp P.H., Osterhaus S.R., Jiang P., Wohlford-Lenane C., Lennox K.A., Jacobi A.M., Praekh K., Rose S.D., Behlke M.A. (2013). Post-transcriptional regulation of cystic fibrosis transmembrane conductance regulator expression and function by microRNAs. Am. J. Respir. Cell Mol. Biol..

[56] Amato F., Seia M., Giordano S., Elce A., Zarrilli F., Castaldo G., Tomaiuolo R. (2013). Gene Mutation in MicroRNA Target Sites of CFTR Gene: A Novel Pathogenetic Mechanism in Cystic Fibrosis?. PLoS ONE.

[57] Sonneville F., Ruffin M., Coraux C., Rousselet N., Le Rouzic P., Blouquit-Laye S., Corvol H., Tabary O. (2017). MicroRNA-9 downregulates the ANO1 chloride channel and contributes to cystic fibrosis lung pathology. Nat. Commun..

[58] Ramachandran S., Karp P.H., Jiang P., Ostedgaard L.S., Walz A.E., Fisher J.T., Keshavjee S., Lennox K.A., Jacobi A.M., Rose S.D. (2012). A microRNA network regulates expression and biosynthesis of wild-type and ΔF508 mutant cystic fibrosis transmembrane conductance regulator. PNAS.

[59] Benedetto R., Ousingsawat J., Wanitchakool P., Zhang Y., Holtzman M.J., Amaral M., Rock J.R., Schreiber R., Kunzelmann K. (2017). Epithelial Chloride Transport by CFTR Requires TMEM16A. Sci. Rep..

[60] Oglesby I.K., Chotirmall S.H., McElvaney N.G., Greene C.M. (2013). Regulation of cystic fibrosis transmembrane conductance regulator by microRNA-145, -223, and -494 is altered in ΔF508 cystic fibrosis airway epithelium. J. Immunol..

[61] Lutful Kabir F., Ambalavanan N., Liu G., Li P., Solomon G.M., Lal C.V., Mazur M., Halloran B., Szul T., Gerthoffer W.T. (2018). MicroRNA-145 Antagonism Reverses TGF-β Inhibition of F508del CFTR Correction in Airway Epithelia. Am. J. Respir. Crit. Care Med..

[62] Mitash N., Mu F., Donovan J.E., Myerburg M.M., Ranganathan S., Greene C.M., Swiatecka-Urban A. (2019). Transforming Growth Factor-β1 Selectively Recruits microRNAs to the RNA-Induced Silencing Complex and Degrades CFTR mRNA under Permissive Conditions in Human Bronchial Epithelial Cells. Int. J. Mol. Sci..

[63] Fenker D.E., McDaniel C.T., Panmanee W., Panos R.J., Sorscher E.J., Sabusap C., Clancy J.P., Hassett D.J. (2018). A Comparison between Two Pathophysiologically Different yet Microbiologically Similar Lung Diseases: Cystic Fibrosis and Chronic Obstructive Pulmonary Disease. Int. J. Respir. Pulm. Med..

[64] Dutta R.K., Chinnapaiyan S., Rasmussen L., Raju S.V., Unwalla H.J. (2019). A Neutralizing Aptamer to TGFBR2 and miR-145 Antagonism Rescue Cigarette Smoke- and TGF-β-Mediated CFTR Expression. Mol. Ther..

[65] Villella V.R., Esposito S., Ferrari E., Monzani R., Tosco A., Rossin F., Castaldo A., Silano M., Marseglia G.L., Romani L. (2019). Autophagy suppresses the pathogenic immune response to dietary antigens in cystic fibrosis. Cell Death Dis..

[66] Maiuri M.C., Kroemer G. (2019). Therapeutic modulation of autophagy: Which disease comes first?. Cell Death Differ..

[67] Tazi M.F., Dakhlallah D.A., Caution K., Gerber M.M., Chang S.-W., Khalil H., Kopp B.T., Ahmed A.E., Krause K., Davis I. (2016). Elevated Mirc1/Mir17-92 cluster expression negatively regulates autophagy and CFTR (cystic fibrosis transmembrane conductance regulator) function in CF macrophages. Autophagy.

[68] Roesch E.A., Nichols D.P., Chmiel J.F. (2018). Inflammation in cystic fibrosis: An update. Pediatr. Pulmonol..

[69] Serhan C.N. (2014). Pro-resolving lipid mediators are leads for resolution physiology. Nature.

[70] Karp C.L., Flick L.M., Park K.W., Softic S., Greer T.M., Keledjian R., Yang R., Uddin J., Guggino W.B., Atabani S.F. (2004). Defective lipoxin-mediated anti-inflammatory activity in the cystic fibrosis airway. Nat. Immunol..

[71] Pierdomenico A.M., Patruno S., Codagnone M., Simiele F., Mari V.C., Plebani R., Recchiuti A., Romano M. (2017). microRNA-181b is increased in cystic fibrosis cells and impairs lipoxin A 4 receptor-dependent mechanisms of inflammation resolution and antimicrobial defense. Sci. Rep..

[72] Luly F.R., Lévêque M., Licursi V., Cimino G., Martin-Chouly C., Théret N., Negri R., Cavinato L., Ascenzioni F., Del Porto P. (2019). MiR-146a is over-expressed and controls IL-6 production in cystic fibrosis macrophages. Sci. Rep..

[73] Jundi K., Greene C.M. (2015). Transcription of Interleukin-8: How Altered Regulation Can Affect Cystic Fibrosis Lung Disease. Biomolecules.

[74] Guan X., Hou Y., Sun F., Yang Z., Li C. (2016). Dysregulated Chemokine Signaling in Cystic Fibrosis Lung Disease: A Potential Therapeutic Target. Curr. Drug. Targets.

[75] Oglesby I.K., Vencken S.F., Agrawal R., Gaughan K., Molloy K., Higgins G., McNally P., McElvaney N.G., Mall M.A., Greene C.M. (2015). miR-17 overexpression in cystic fibrosis airway epithelial cells decreases interleukin-8 production. Eur. Respir. J..

[76] Fabbri E., Borgatti M., Montagner G., Bianchi N., Finotti A., Lampronti I., Bezzerri V., Dechecchi M.C., Cabrini G., Gambari R. (2014). Expression of microRNA-93 and Interleukin-8 during Pseudomonas aeruginosa–Mediated Induction of Proinflammatory Responses. Am. J. Respir. Cell Mol. Biol..

[77] Bhattacharyya S., Balakathiresan N.S., Dalgard C., Gutti U., Armistead D., Jozwik C., Srivastava M., Pollard H.B., Biswas R. (2011). Elevated miR-155 promotes inflammation in cystic fibrosis by driving hyperexpression of interleukin-8. J. Biol. Chem..

[78] Bhattacharyya S., Kumar P., Tsuchiya M., Bhattacharyya A., Biswas R. (2013). Regulation of miR-155 biogenesis in cystic fibrosis lung epithelial cells: Antagonistic role of two mRNA-destabilizing proteins, KSRP and TTP. Biochem. Biophys. Res. Commun..

[79] Tsuchiya M., Kalurupalle S., Kumar P., Ghoshal S., Zhang Y., Lehrmann E., Becker K.G., Gorospe M., Biswas R. (2016). RPTOR, a novel target of miR-155, elicits a fibrotic phenotype of cystic fibrosis lung epithelium by upregulating CTGF. RNA Biol..

[80] Bardin P., Marchal-Duval E., Sonneville F., Blouquit-Laye S., Rousselet N., Rouzic P.L., Corvol H., Tabary O. (2018). Small RNA and transcriptome sequencing reveal the role of miR-199a-3p in inflammatory processes in cystic fibrosis airways. J. Pathol..

[81] Zhang P., Cheng J., Zou S., D’Souza A.D., Koff J.L., Lu J., Lee P.J., Krause D.S., Egan M.E., Bruscia E.M. (2015). Pharmacological modulation of the AKT/microRNA-199a-5p/CAV1 pathway ameliorates cystic fibrosis lung hyper-inflammation. Nat. Commun..

[82] Ge Q., Moir L.M., Black J.L., Oliver B.G., Burgess J.K. (2010). TGFβ1 induces IL-6 and inhibits IL-8 release in human bronchial epithelial cells: The role of Smad2/3. J. Cell. Physiol..

[83] Oglesby I.K., Bray I.M., Chotirmall S.H., Stallings R.L., O’Neill S.J., McElvaney N.G., Greene C.M. (2010). miR-126 Is Downregulated in Cystic Fibrosis Airway Epithelial Cells and Regulates TOM1 Expression. J. Immunol..

[84] Stolzenburg L.R., Wachtel S., Dang H., Harris A. (2016). miR-1343 attenuates pathways of fibrosis by targeting the TGF-β receptors. Biochem. J..

[85] Zhong T., Perelman J.M., Kolosov V.P., Zhou X. (2011). MiR-146a negatively regulates neutrophil elastase-induced MUC5AC secretion from 16HBE human bronchial epithelial cells. Mol. Cell. Biochem..

[86] Ribeiro C.M.P., Boucher R.C. (2010). Role of Endoplasmic Reticulum Stress in Cystic Fibrosis–Related Airway Inflammatory Responses. Proc. Am. Thorac. Soc..

[87] Oglesby I.K., Agrawal R., Mall M.A., McElvaney N.G., Greene C.M. (2015). miRNA-221 is elevated in cystic fibrosis airway epithelial cells and regulates expression of ATF6. Mol. Cell. Pediatr..

[88] Weldon S., McNally P., McAuley D.F., Oglesby I.K., Wohlford-Lenane C.L., Bartlett J.A., Scott C.J., McElvaney N.G., Greene C.M., McCray P.B. (2014). miR-31 dysregulation in cystic fibrosis airways contributes to increased pulmonary cathepsin S production. Am. J. Respir. Crit. Care Med..

[89] Brown R., Nath S., Lora A., Samaha G., Elgamal Z., Kaiser R., Taggart C., Weldon S., Geraghty P. (2020). Cathepsin S: Investigating an old player in lung disease pathogenesis, comorbidities, and potential therapeutics. Respir. Res..

[90] Velu V.K., Ramesh R., Srinivasan A.R. (2012). Circulating MicroRNAs as Biomarkers in Health and Disease. J. Clin. Diagn. Res..

[91] Wang H., Peng R., Wang J., Qin Z., Xue L. (2018). Circulating microRNAs as potential cancer biomarkers: The advantage and disadvantage. Clin. Epigenetics.

[92] Russo F., Scoyni F., Fatica A., Pellegrini M., Ferro A., Pulvirenti A., Giugno R., García-Giménez J.L. (2016). Chapter 12—Circulating Noncoding RNAs as Clinical Biomarkers. Epigenetic Biomarkers and Diagnostics.

[93] Gibbings D.J., Ciaudo C., Erhardt M., Voinnet O. (2009). Multivesicular bodies associate with components of miRNA effector complexes and modulate miRNA activity. Nat. Cell Biol..

[94] Lim S.B., Di Lee W., Vasudevan J., Lim W.-T., Lim C.T. (2019). Liquid biopsy: One cell at a time. NPJ Precis. Oncol..

[95] Krause K., Kopp B.T., Tazi M.F., Caution K., Hamilton K., Badr A., Shrestha C., Tumin D., Hayes D., Robledo-Avila F. (2018). The expression of Mirc1/Mir17–92 cluster in sputum samples correlates with pulmonary exacerbations in cystic fibrosis patients. J. Cyst. Fibros..

[96] Ideozu J.E., Zhang X., Rangaraj V., McColley S., Levy H. (2019). Microarray profiling identifies extracellular circulating miRNAs dysregulated in cystic fibrosis. Sci. Rep..

[97] Chotirmall S.H., Greene C.M., Harvey B.J., McElvaney N.G., Sriramulu D. (2012). The Cystic Fibrosis “Gender Gap”: Past Observations Present Understanding and Future Directions. Cystic Fibrosis—Renewed Hopes Through Research.

[98] Saint-Criq V., Harvey B.J. (2014). Estrogen and the cystic fibrosis gender gap. Steroids.

[99] Mooney C., McKiernan P.J., Raoof R., Henshall D.C., Linnane B., McNally P., Glasgow A.M.A., Greene C.M. (2020). Plasma microRNA levels in male and female children with cystic fibrosis. Sci. Rep..

[100] Cook N.L., Pereira T.N., Lewindon P.J., Shepherd R.W., Ramm G.A. (2015). Circulating microRNAs as noninvasive diagnostic biomarkers of liver disease in children with cystic fibrosis. J. Pediatr. Gastroenterol. Nutr..

[101] Baldassarre A., Felli C., Prantera G., Masotti A. (2017). Circulating microRNAs and Bioinformatics Tools to Discover Novel Diagnostic Biomarkers of Pediatric Diseases. Genes.

[102] Calvopina D.A., Chatfield M.D., Weis A., Coleman M.A., Fernandez-Rojo M.A., Noble C., Ramm L.E., Leung D.H., Lewindon P.J., Ramm G.A. (2018). MicroRNA Sequencing Identifies a Serum MicroRNA Panel, Which Combined With Aspartate Aminotransferase to Platelet Ratio Index Can Detect and Monitor Liver Disease in Pediatric Cystic Fibrosis. Hepatology.

[103] Montanini L., Smerieri A., Gullì M., Cirillo F., Pisi G., Sartori C., Amarri S., Bernasconi S., Marmiroli N., Street M.E. (2016). miR-146a, miR-155, miR-370, and miR-708 Are CFTR-Dependent, Predicted FOXO1 Regulators and Change at Onset of CFRDs. J. Clin. Endocrinol. Metab..

[104] Zhang X., Pan A., Jia S., Ideozu J.E., Woods K., Murkowski K., Hessner M.J., Simpson P.M., Levy H. (2019). Cystic Fibrosis Plasma Blunts the Immune Response to Bacterial Infection. Am. J. Respir. Cell Mol. Biol..

[105] Staufer K. (2020). Current Treatment Options for Cystic Fibrosis-Related Liver Disease. Int. J. Mol. Sci..

[106] Leeuwen L., Fitzgerald D.A., Gaskin K.J. (2014). Liver disease in cystic fibrosis. Paediatr. Respir. Rev..

[107] Betapudi B., Aleem A., Kothadia J.P. (2020). Cystic Fibrosis and Liver Disease. StatPearls.

[108] Lewindon P.J., Shepherd R.W., Walsh M.J., Greer R.M., Williamson R., Pereira T.N., Frawley K., Bell S.C., Smith J.L., Ramm G.A. (2011). Importance of hepatic fibrosis in cystic fibrosis and the predictive value of liver biopsy. Hepatology.

[109] Ahanda M.-L.E., Bienvenu T., Sermet-Gaudelus I., Mazzolini L., Edelman A., Zoorob R., Davezac N. (2015). The hsa-miR-125a/hsa-let-7e/hsa-miR-99b cluster is potentially implicated in Cystic Fibrosis pathogenesis. J. Cyst. Fibros..

[110] Chakraborty C., Sharma A.R., Sharma G., Doss C.G.P., Lee S.-S. (2017). Therapeutic miRNA and siRNA: Moving from Bench to Clinic as Next Generation Medicine. Mol. Nucleic Acids.

[111] Lima J.F., Cerqueira L., Figueiredo C., Oliveira C., Azevedo N.F. (2018). Anti-miRNA oligonucleotides: A comprehensive guide for design. RNA Biol..

[112] Ebert M.S., Neilson J.R., Sharp P.A. (2007). MicroRNA sponges: Competitive inhibitors of small RNAs in mammalian cells. Nat. Methods.

[113] Gumireddy K., Young D.D., Xiong X., Hogenesch J.B., Huang Q., Deiters A. (2008). Small Molecule Inhibitors of MicroRNA miR-21 Function. Angew. Chem. Int. Ed. Engl..

[114] Chan J.H.P., Lim S., Wong W.S.F. (2006). Antisense oligonucleotides: From design to therapeutic application. Clin. Exp. Pharm. Physiol..

[115] Bartoszewski R., Sikorski A.F. (2019). Editorial focus: Understanding off-target effects as the key to successful RNAi therapy. Cell. Mol. Biol. Lett..

[116] Wei T., Sui H., Su Y., Cheng W., Liu Y., He Z., Ji Q., Xu C. (2020). Research advances in molecular mechanisms underlying the pathogenesis of cystic fibrosis: From technical improvement to clinical applications (Review). Mol. Med. Rep..

[117] Bardin P., Sonneville F., Corvol H., Tabary O. (2018). Emerging microRNA Therapeutic Approaches for Cystic Fibrosis. Front. Pharm..

[118] McKiernan P.J., Cunningham O., Greene C.M., Cryan S.-A. (2013). Targeting miRNA-based medicines to cystic fibrosis airway epithelial cells using nanotechnology. Int. J. Nanomed..

[119] Amato F., Tomaiuolo R., Borbone N., Elce A., Amato J., D’Errico S., Rosa G.D., Mayol L., Piccialli G., Oliviero G. (2014). Design, synthesis and biochemical investigation, by in vitro luciferase reporter system, of peptide nucleic acids as new inhibitors of miR-509-3p involved in the regulation of cystic fibrosis disease-gene expression. MedChemComm.

[120] Amato F., Tomaiuolo R., Nici F., Borbone N., Elce A., Catalanotti B., D’Errico S., Morgillo C.M., De Rosa G., Mayol L. Exploitation of a Very Small Peptide Nucleic Acid as a New Inhibitor of miR-509-3p Involved in the Regulation of Cystic Fibrosis Disease-Gene Expression. https://www.hindawi.com/journals/bmri/2014/610718/.

[121] Fabbri E., Tamanini A., Jakova T., Gasparello J., Manicardi A., Corradini R., Sabbioni G., Finotti A., Borgatti M., Lampronti I. (2017). A Peptide Nucleic Acid against MicroRNA miR-145-5p Enhances the Expression of the Cystic Fibrosis Transmembrane Conductance Regulator (CFTR) in Calu-3 Cells. Molecules.

[122] Fabbri E., Tamanini A., Jakova T., Gasparello J., Manicardi A., Corradini R., Finotti A., Borgatti M., Lampronti I., Munari S. (2020). Treatment of human airway epithelial Calu-3 cells with a peptide-nucleic acid (PNA) targeting the microRNA miR-101-3p is associated with increased expression of the cystic fibrosis Transmembrane Conductance Regulator () gene. Eur. J. Med. Chem..

[123] Sultan S., Rozzi A., Gasparello J., Manicardi A., Corradini R., Papi C., Finotti A., Lampronti I., Reali E., Cabrini G. (2020). A Peptide Nucleic Acid (PNA) Masking the miR-145-5p Binding Site of the 3′UTR of the Cystic Fibrosis Transmembrane Conductance Regulator (CFTR) mRNA Enhances CFTR Expression in Calu-3 Cells. Molecules.

[124] Zarrilli F., Amato F., Morgillo C.M., Pinto B., Santarpia G., Borbone N., D’Errico S., Catalanotti B., Piccialli G., Castaldo G. (2017). Peptide Nucleic Acids as miRNA Target Protectors for the Treatment of Cystic Fibrosis. Molecules.

[125] Finotti A., Gasparello J., Fabbri E., Tamanini A., Corradini R., Dechecchi M.C., Cabrini G., Gambari R. (2019). Enhancing the Expression of CFTR Using Antisense Molecules against MicroRNA miR-145-5p. Am. J. Respir. Crit. Care Med..

[126] Santi C.D., Fernández E.F., Gaul R., Vencken S., Glasgow A., Oglesby I.K., Hurley K., Hawkins F., Mitash N., Mu F. (2020). Precise Targeting of miRNA Sites Restores CFTR Activity in CF Bronchial Epithelial Cells. Mol. Ther..

[127] McCarron A., Parsons D., Donnelley M. (2020). Animal and Cell Culture Models for Cystic Fibrosis: Which Model Is Right for Your Application?. Am. J. Pathol..

[128] Semaniakou A., Croll R.P., Chappe V. (2019). Animal Models in the Pathophysiology of Cystic Fibrosis. Front. Pharm..

[129] Chakradhar S. (2017). Put to the test: Organoid-based testing becomes a clinical tool. Nat. Med..

[130] Dekkers J.F., Wiegerinck C.L., de Jonge H.R., Bronsveld I., Janssens H.M., de Winter-de Groot K.M., Brandsma A.M., de Jong N.W.M., Bijvelds M.J.C., Scholte B.J. (2013). A functional CFTR assay using primary cystic fibrosis intestinal organoids. Nat. Med..

